# A review and comparative study of cancer detection using machine learning: SBERT and SimCSE application

**DOI:** 10.1186/s12859-023-05235-x

**Published:** 2023-03-23

**Authors:** Mpho Mokoatle, Vukosi Marivate, Darlington Mapiye, Riana Bornman, Vanessa. M. Hayes

**Affiliations:** 1 Department of Computer Science, University of Pretoria, Pretoria, South Africa; 2 CapeBio TM Technologies, Centurion, South Africa; 3 School of Medical Sciences, The University of Sydney, Sydney, Australia; 4 School of Health Systems and Public Health, University of Pretoria, Pretoria, South Africa

**Keywords:** Cancer detection, DNA, Machine learning, SentenceBert, SimCSE

## Abstract

**Background:**

Using visual, biological, and electronic health records data as the sole input source, pretrained convolutional neural networks and conventional machine learning methods have been heavily employed for the identification of various malignancies. Initially, a series of preprocessing steps and image segmentation steps are performed to extract region of interest features from noisy features. Then, the extracted features are applied to several machine learning and deep learning methods for the detection of cancer.

**Methods:**

In this work, a review of all the methods that have been applied to develop machine learning algorithms that detect cancer is provided. With more than 100 types of cancer, this study only examines research on the four most common and prevalent cancers worldwide: lung, breast, prostate, and colorectal cancer. Next, by using state-of-the-art sentence transformers namely: SBERT (2019) and the unsupervised SimCSE (2021), this study proposes a new methodology for detecting cancer. This method requires raw DNA sequences of matched tumor/normal pair as the only input. The learnt DNA representations retrieved from SBERT and SimCSE will then be sent to machine learning algorithms (XGBoost, Random Forest, LightGBM, and CNNs) for classification. As far as we are aware, SBERT and SimCSE transformers have not been applied to represent DNA sequences in cancer detection settings.

**Results:**

The XGBoost model, which had the highest overall accuracy of 73 ± 0.13 % using SBERT embeddings and 75 ± 0.12 % using SimCSE embeddings, was the best performing classifier. In light of these findings, it can be concluded that incorporating sentence representations from SimCSE’s sentence transformer only marginally improved the performance of machine learning models.

**Supplementary Information:**

The online version contains supplementary material available at 10.1186/s12859-023-05235-x.

## Introduction

Cancer is a disease where some cells in the body grow destructively and may spread to other body organs [1]. Typically, cells grow and expand through a cell division process to create new cells that can be used to repair old and damaged ones. However, this phenomenon can be interrupted resulting in abnormal cells growing uncontrollably to form tumors that can be malignant (harmful) or benign (harmless) [2–4].

With the introduction of genomic data that allows physicians and healthcare decision-makers to learn more about their patients and their response to the therapy they provide to them, this has facilitated the use of machine learning and deep learning to solve challenging cancer problems. These kinds of problems involve various tasks such as designing cancer risk-prediction models that try to identify patients that are at a higher risk of developing cancer than the general population, studying the progression of the disease to improve survival rates, and building methods that trace the effectiveness of treatment to improve treatment options [5–7].

Generally, the first step in analyzing genomic data to address cancer-related problems is selecting a data representation algorithm that will be used to estimate contiguous representations of the data. Examples of such algorithms include Word2vec [8], GloVe [9], and fastText [10]. The more recent and advanced versions of these algorithms are sentence transformers which are used to compute dense vector representations for sentences, paragraphs, and images. Similar texts are found close together in a vector space and dissimilar texts are far apart [11]. In this work, two such sentence transformers (SBERT and SimCSE) are proposed for detecting cancer in tumor/normal pairs of colorectal cancer patients. In this new approach, the classification algorithm relies on raw DNA sequences as the only input source. Moreover, this work provides a review of the most recent developments in cancers of the human body using machine learning and deep learning methods. While these kinds of similar reviews already exist in the literature, this study solely focuses on work that investigates four cancer types that have high prevalence rates worldwide [12] (lung, breast, prostate, and colorectal cancer) that have been published in the last five years (2018–2022).

## Detection of cancer using machine learning

### Lung cancer

Lung cancer is the type of cancer that begins in the lungs and may spread to other organs in the body. This kind of cancer occurs when malignant cells develop in the tissue of the lung. There are two types of lung cancer: non-small-cell lung cancer (NSCLC) and small-cell lung cancer (SCLC). These cancers develop differently and thus their treatment therapies are different. Smoking (tobacco) is the leading cause of lung cancer. However, non-smokers can also develop lung cancer [13, 14].

**Fig. 1:**
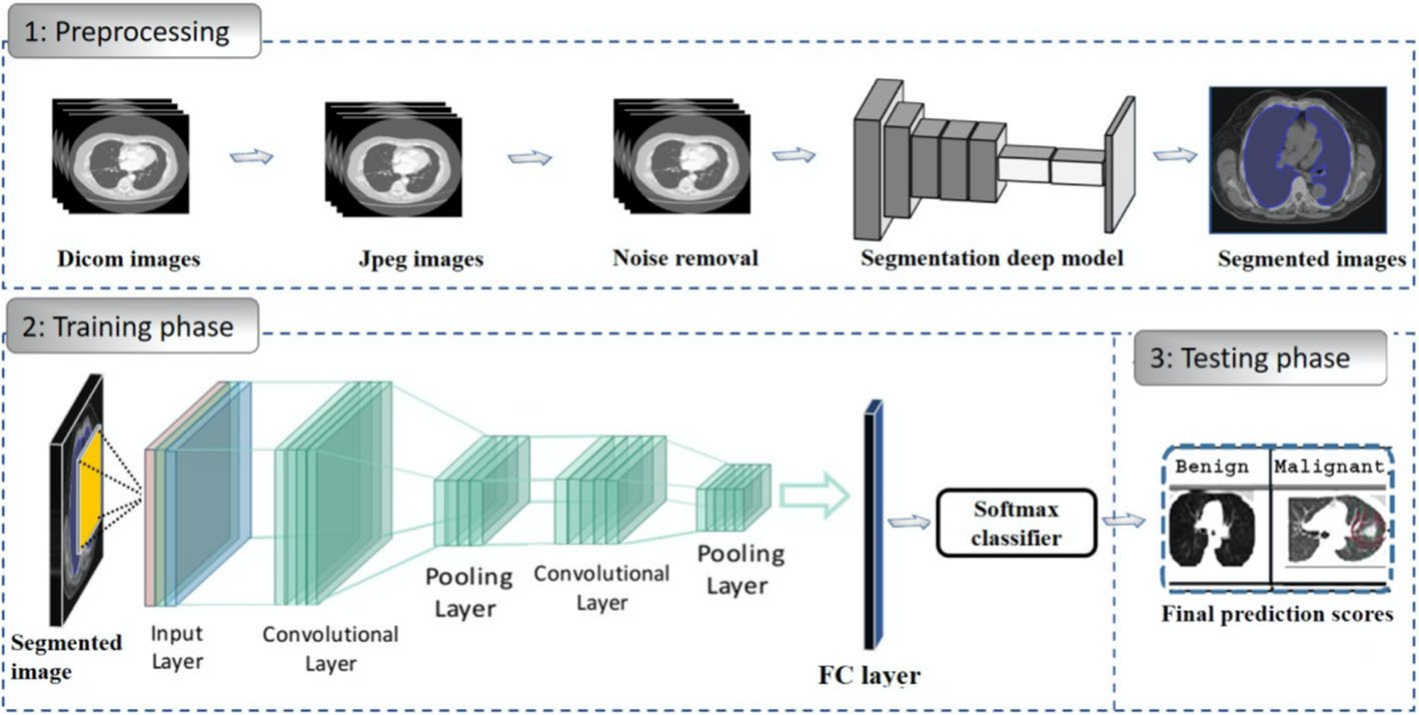
Generalized machine learning framework for lung cancer prediction [33]

When it comes to the detection of lung cancer using machine learning (Fig. 1), a considerable amount of work has been done, a summary is provided (Table 1). Typically, a series of pre-processing steps using statistical methods and pretrained CNNs for feature extraction are carried out from several input sources (mostly images) to delineate the cancer region. Then, the extracted features are fed as input to several machine learning algorithms for classification of various lung cancer tasks such as the detection of malignant lung nodules from benign ones [15–17], the separation of a set of normalized biological data points into cancerous and non cancerous groups [18], and a basic comparative analysis of powerful machine learning algorithms for lung cancer detection [19].

**Table 1:** This table gives a summary of recent work that has been performed in lung cancer detection using machine learning and deep learning algorithms as discussed in Sect. 2.1

References	Feature extraction	Data	ML/DL	Acc (%)
[15] 2018	taxic weights, phylogenetic trees	LIDC-IDRI [23]	CNNs	92.6
[16] 2018	SCM	LIDC-IDRI [23]	MLP, $k-$NN, SVM	96.7
[20] 2018	histogram equalization	JSRT [22], ChestX-ray14 [21]	DenseNet	74.4
[25] 2019	–	UCI [26]	SVM,LR,DT,Naive Bayes	99.2
[18] 2019	AdaBoost	ELVIRA biomedical data [27]	ANN	99.7
[28] 2019	UNet and ResNet	LIDC-IDRI [23]	XGBoost and RF	84.0
[29] 2020	–	spectroscopic data	ResNet	95.0
[17] 2020	HoG, LBP, SIFT, Zernike Moment	LIDC-IDRI [23]	FPSOCNN	95.6
[30] 2021	2D-DFT and 2D-DWT	LC25000 images [24]	CNNs	96.3
[19] 2021	Correlation Attribute (CA)	UCI [26]	CNN, SVM, *k*-NN	95.5
[31] 2022	LeNet, AlexNet, VGG16, ResNet-50, Inception-V1	LUNA16 [32]	Fully connected layer	97.25

The lowest classification accuracy reported in Table 1 was 74.4% by work in [20]. In this work, a pretrained CNN model (DenseNet) was used to develop a lung cancer detection model. First, the model was fine-tuned to identify lung nodules from chest X-rays using the ChestX-ray14 dataset [21]. Second, the model was fine-tuned to identify lung cancer from images in the JSRT (Japanese Society of Radiological Technology) dataset [22].

The highest classification accuracy of 99.7% for lung cancer classification was reported by work in [18]. This study developed the Discrete AdaBoost Optimized Ensemble Learning Generalized Neural Network (DAELGNN) framework that uses a set of normalized biological data points to create a neural network that separates normal lung features from non-normal (cancerous) features.

Popular datasets used in lung cancer research using machine learning include the Lung Image Database Consortium (LIDC) and Image Database Resource Initiative (IDRI) (LIDC-IDRI) database [23] initiated by the National Cancer Institute (NCI), and the histopathological images of lung and colon cancer (LC2500) database [24].

### Breast cancer

Breast Cancer is a malignant tumor or growth that develops in the cells of the breast [34]. Similar to lung cancer, breast cancer also has the ability to metastasize to near by lymph nodes or to other body organs. Towards the end of 2020, there were approximately 7.8 million women who have been diagnosed with breast cancer, making this type of cancer the most prevalent cancer in the world. Risk factors of breast cancer include age, obesity, abuse of alcohol, and family history [35–37].

Currently, there is no identified prevention procedure for breast cancer. However, maintaining a healthy living habit such as physical exercise and less alcohol intake can reduce the risk of developing breast cancer [38]. It has also been said that early detection methods that rely on machine learning can improve the prognosis. As such, this type of cancer has been extensively studied using machine learning and deep learning [39, 40].

**Fig. 2:**
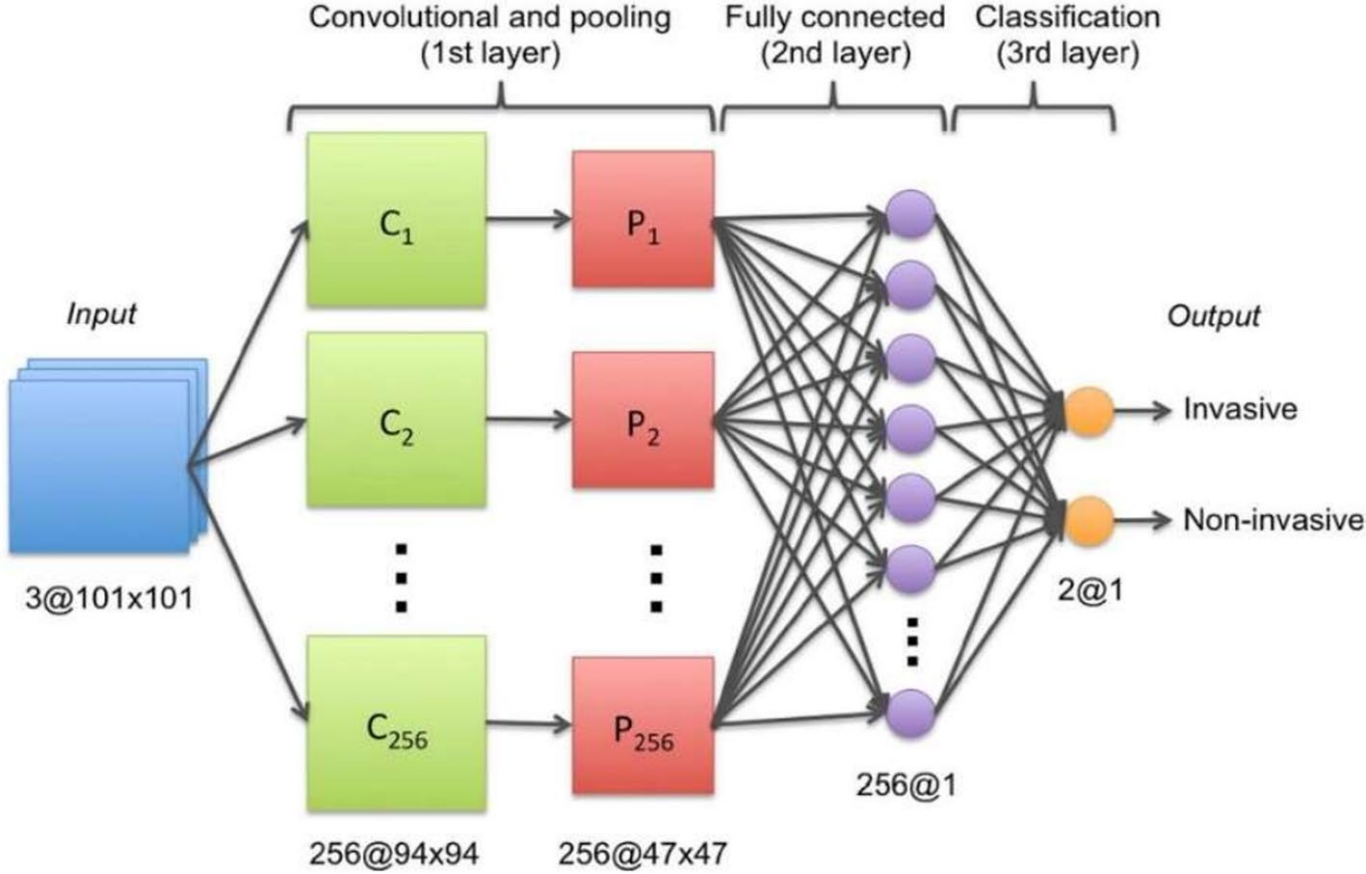
Generalized machine learning framework for breast cancer prediction [45]

As with lung cancer (Sect. 2.1), a great deal of work has been executed in developing breast cancer detection models, a generalized approach that illustrates the process using machine learning is provided (Fig. 2).

Several classification problems have been studied that mainly focuses on the detection of breast cancer from thermogram images [41], handrafted features [42], mammograms [43], and whole slide images [44]. To develop a breast cancer detection model, initially, a pre-processing step is implemented that aims to extract features of interest. Then, the extracted features are provided as input to machine learning models for classification. This framework is implemented by several works such as [45–48].

One of the most popular datasets used for breast cancer detection using machine learning is the Wisconsin breast cancer dataset [42]. This dataset consists of features that describe the characteristics of the cell nuclei that is present in the image such as the diagnosis features (malignant or benign), radius, symmetry, and texture. Studies that used this dataset are [49, 50]. In [49], the authors scaled the Wisconsin breast cancer features to be in the range between 0 and 1, then used a CNN for classification into benign or malignant. As opposed to using a CNN for classification, the authors [50] used traditional machine learning classifiers (Linear Regression, Multilayer Perceptron (MLP), Nearest Neighbor search, Softmax Regression, Gated recurrent Unit (GRU)-SVM, and SVM). For data pre-processing, the study used the Standard Scaler technique that standardizes data points by removing the mean and scaling the data to unit variance. The MLP model outperformed the other models by producing the highest accuracy of 99.04% which is almost similar to the accuracy of 99.6% that was reported by [49].

Different form binary classification of benign or malignant classes, a study [46] proposed a two-step approach to design a breast cancer multi-class classification model that predicts eight categories of breast cancer. In the first approach, the study used handcrafted features that are generated from histopathology images. These features were then fed as input to classical machine learning algorithms (RF, SVM, Linear Discriminant Analysis (LDA)). In the second approach, the study applied a transfer learning method to develop the multi-classification deep learning framework where pretained CNNs (ResNet50, VGG16 and VGG19) were used as feature extractors and baseline models. It was then found that the VGG16 pretrained CNN with the linear SVM provided the best accuracy in the range of 91.23%$-$93.97%. This study also found that using pretrained CNNs as feature extractors improved the classification performance of the models.

The Table 2 provides a summary of the work that has been done to detect breast cancer using machine learning.

**Table 2:** This table gives a summary of recent work that has been executed in breast cancer detection using machine learning and deep learning algorithms as discussed in Sect. 2.2

References	Feature extraction	Data	ML/DL	Acc, AUC or ROC (%)
[49] 2018	Watershed Segmentation	histopathology images	CNN	98
[49] 2018	Label encoder, normalization	Wisconsin breast cancer [42]	CNN	99.6
[50] 2018	Standard scaler	Wisconsin breast cancer [42]	GRU-SVM, Linear Regression,	
			MLP, Nearest Neighbor,	
			Softmax Regression, SVM	99.0
[45] 2018	Inception V3	thermogram images [41]	LinearSVC, SVM	100
[51] 2019	–	[52] CBIS-DDSM, [53] INbreast	ResNet50, VGG16	65-97
[48] 2020	Histogram-sigmoid fuzzy clustering	histopathology images	Deep Neural Network	97
[44] 2019	filters	whole slide images	CNN	88
[46] 2020	Hu moment, color histogram,			
	and Haralick textures, ResNet50,			
	VGG16 and VGG19	BreakHis [54]	RF, SVM, LDA,	
			ResNet50, VGG16, VGG19	91.2-93.9
[55] 2021	–	IDC patch images [56]	CNNs,LR,SVM, KNN	87
[47] 2022	AWS, DenseNet-169	mammograms [43]	MLP	93.8
[57] 2022	AlexNet CNN	ultrasound images and histopathological images	Fully connected layer	96.7-100
[58] 2022	AlexNet CNN	MRI scans [59]	Fully connected layer	98.1-98.44
[60] 2022	–	Wisconsin Breast Cancer Diagnostic data	deep extreme gradient descent optimization	98.73

### Prostate cancer

Prostate cancer is a type of cancer that develops when cells in the prostate gland start to grow uncontrollably (malignant). Prostate cancer often presents with no symptoms and grows at a slow rate. As a result, some men may die of other diseases before the cancer starts to cause notable problems. Comparably, prostate cancer can also be aggressive and metastasize to other body organs that are outside the confines of the prostate gland. Risk factors that are associated with this type of cancer include age, specifically, men that are above the age of 50. Other risk factors include ethnicity, family history of prostate cancer, breast or ovarian cancer, and obesity [61–63].

**Fig. 3:**
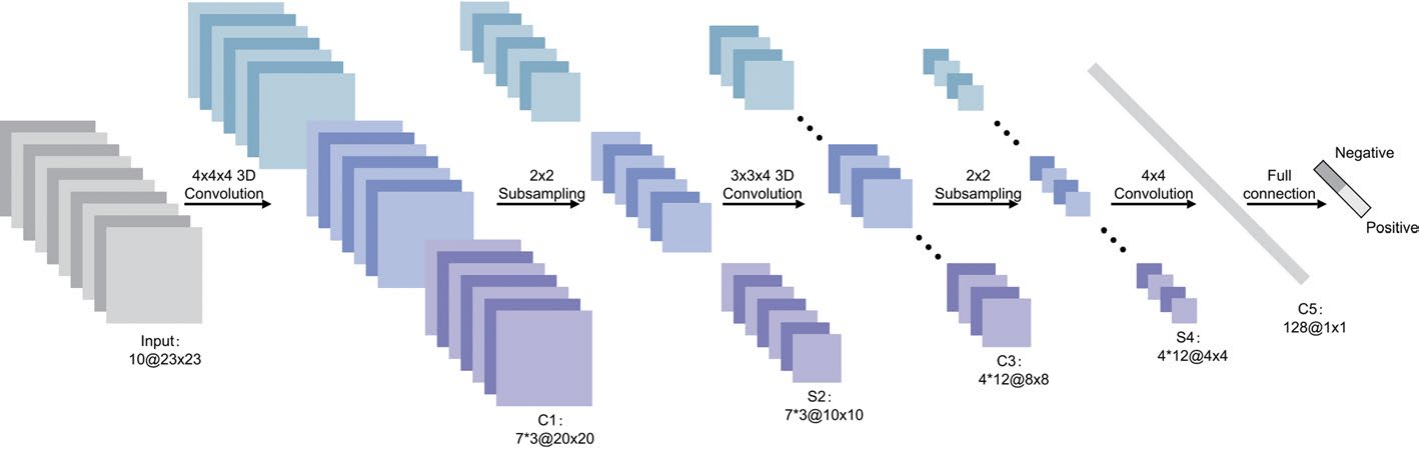
Generalized machine learning framework for prostate cancer prediction using 3-d CNNs, pooling layers, and a fully connected layer for classification [69]

Transfer learning, which is defined as the reuse of a pretrained model on a new problem, was frequently applied to develop prostate cancer detection models using machine learning (Fig. 3). For example, a study [64] applied a transfer learning approach to detect prostate cancer on magnetic resonance images (MRI) by using a pretrained GoogleNet. A series of features such as texture, entropy, morphological, scale invariant feature transform (SIFT), and Elliptic Fourier Descriptors (EFDs) were extracted from the images as described by [65, 66]. Other traditional machine learning classifiers were also evaluated such as Decision trees, and SVM Gaussian however, the GoogleNet model outperformed the other models.

Also using transfer learning, a study [67] developed a prostate cancer detection model by using MRI images and ultrasound (US) images. The model was developed in two stages: first, pretrained CNNs were used for classification of the US and MRI images into benign or malignant. While the pretrained CNNs performed well on the US images (accuracy 97%), the performance on the MRI images was not adequate. As a result, the best-performing pretrained CNN(VGG16) was selected and used as a feature extractor. The extracted features were then provided as input to traditional machine learning classifiers.

Another study [68] also used the same dataset as in [64] to create a prostate cancer detection model. However, instead of using GoogleNet as seen previously by [64], this study used a ResNet-101 and an autoencoder for feature reduction. Other machine learning models were also evaluated but, the study concluded that the pretrained ResNet-101 outperformed the other models with an accuracy of 100%. These results are similar to a previous study [64] that showed how pretrained CNNs outperform traditional machine learning models for cancer detection.

Table 3, gives a summary of recent work that has been executed to create prostate cancer detection models.

**Table 3:** This table gives a summary of recent work that has been executed in prostate cancer detection using machine learning and deep learning algorithms as discussed in Sect. 2.3

References	Feature extraction	Data	ML/DL	Acc, AUC, or ROC (%)
[69] 2018	3-D CNN	images from CEUS videos	3-D CNN, J48, logistic, RF,	
			Decision Table, FLDA, KNN	90
[70] 2018	level set-based approach, GGMRF	DWI images	SNCSAE, RF, Random Tree,	94
[71] 2019	normalization and scaling	NCI PLCO	KNN, SVM, DT, RF, MLP,	
			Adaptive boosting, Quadratic discriminant analysis	91
[72] 2019	modified ResNet, DT	DWI images	RF	87
[73] 2020	patch extraction principle	whole slide images [74, 75]	NASNetLarge	97.3–98
[64] 2020	As described by [65, 66]	MRI images	GoogleNet, Bayes, decision tree,	
			SVM Gaussian, SVM RBF, SVM polynomial	100
[68] 2021	Statistical methods	MRI images	Kernel Naïve Bayes, DTs, SVM-Gaussian,	
			KNN-Cosine, LSTM, RUSBoost Tree	100
[76] 2021	3-D U-Net	bpMRI images	U-Net	85
[67] 2022	VGG16	US and MRI images [77–79]	RF, SVM, Gradient boosting, NN,	
			MobileNetV2, ResNet50V2, Resnet101V2,	
			Resnet152V2, Xception, VGG16, VGG19,	
			InceptionResNetV2, and InceptionV3	88–97
[80] 2022	slide tiling, Otsu’s method [81]	whole slide images, TCGA data [74]	EfficientNetB1	98–99

### Colorectal cancer

Colorectal cancer is a type of cancer that starts in the colon or rectum. The colon and rectum are parts of the human body that make up the large intestine that is part of the digestive system. A large part of the large intestine is made up of the colon which is divided into a few parts namely: ascending colon, transverse colon, descending colon, and sigmoid colon. The main function of the colon is to absorb water and salt from the remaining food waste after it has passed through the small intestine. Then, the waste that is left after passing through the colon goes into the rectum and is stored there until it is passed through the anus. Some colorectal cancers called polyps first develop as growth that can be found in the inner lining of the colon or rectum. Overtime, these polyps can develop into cancer, however, not all of them can be cancerous. Some of the risk factors of colorectal cancer include obesity, lack of exercise, diets that are rich in red meat, smoking, and alcohol [82–84].

**Fig. 4:**
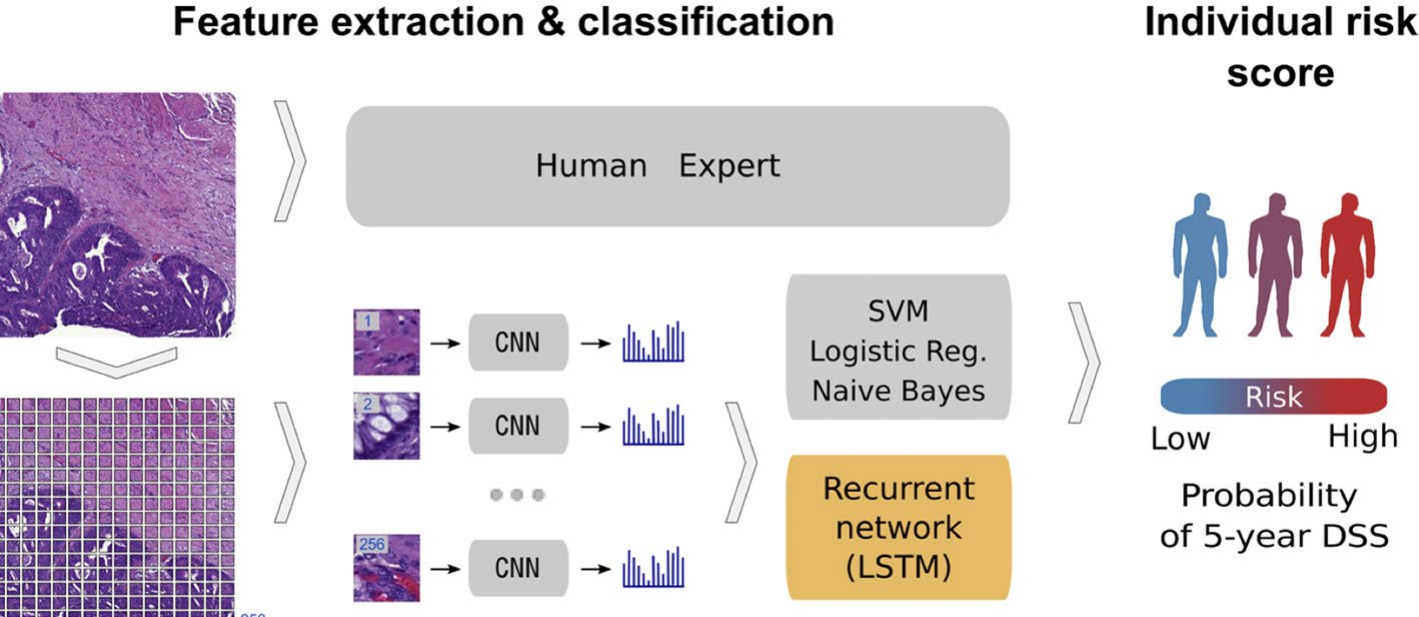
Using a deep CNN network to predict colorectal cancer outcome using images [86]

In relation to the advancements made in colorectal cancer research using machine learning (Fig. 4), various tasks have been investigated such as predicting high-risk colorectal cancer from images, predicting five-year disease-specific survival, colorectal cancer tissue multi-class classification, and identifying the risk factors for lymph node metastasis (LNM) in colorectal cancer patients [85–88]. As with prostate cancer, transfer learning was mostly applied to extract features from various input sources such as colonoscopic images, tissue microarrays (TMA), and H &E slide images. Then, the extracted features were fed as input to machine learning algorithms for classification.

One common observation with regards to colorectal cancer models, is that the predictions made from the models were compared to those of experts. For example, a study [85] developed a deep learning model that detects high risk colorectal cancer from whole slide images that were collected from colon biopsies. The deep learning model was created in two stages: first, a segmentation procedure was executed to extract high risk regions from whole slide images. This segmentation procedure applied Faster-Region Based Convolutional Neural Network (Faster-RCNN) that uses a ResNet-101 model as a backbone for feature extraction. The second stage of implementing the model applied a gradient-boosted decision tree on the output of the Faster-RCNN deep learning model to classify the slides into either high or low risk colorectal cancer, and achieved an AUC of 91.7%. The study then found that the predictions made from the validation set were in agreement with annotations made by expert pathologists.

Work in [89] also compared predictions made by the Microsatellite instability (MSI)-predictor model with those of expert pathologists and found that experts achieved a mean AUROC of 61% while the model achieved an AUROC of 93% on a hold-out set and 87% on a reader experiment.

A previous study [90] developed a model named CRCNet, based a pretrained dense CNN, that automatically detects colorecal cancer from colonoscopic images and found that the model exceeded the avarage performance of expert endoscopists on a recall rate of 91.3% versus 83.8%.

In Table 4, a summary is provided that describes the work that has been executed in colorectal cancer research using machine learning.

**Table 4:** This table gives a summary of recent work that has been executed in colorectal cancer detection/survival using machine learning and deep learning algorithms as discussed in Sect.  2.4

References	Feature extraction	Data	ML/DL	Acc, AUC, ROC, or AUPRC (%)
[86] 2018	VGG16	TMA, Whole slide images	1-d LSTM, SVM, LR, Naive Bayes	61–69
[91] 2019	Normalization	EHR	CNN	92
[92] 2020	Macenko method [93]	H &E slide images [74, 94–97]	ShuffleNet	96
[90] 2020	–	Colonoscopic images	169-layer dense CNN	86.7–88.2
[89] 2021	Thresholding and normalization	Whole slide images [74, 98]	MobileNetV2	78–93
[99] 2021	Contrast-Limited Adaptive			
	Histogram Equalization (CLAHE)	Warwick-QU dataset [100]	ResNet-18 & ResNet-50	73–88
[101] 2021	Normalization and data labeling	Numeric and clinical data	FNNs, SVMs, LR, LDA	77
[85] 2022	Faster-RCNN	Whole slide images	Gradient-boosted decision tree	91
[87] 2021	VGG16	Whole slide images [102]	MLP	99
[88] 2022	Aachen protocol [103] and,			
	Macenko normalisation[93]	Whole slide images and		
		clinical pathological data	ShuffleNet	56–73

In summary of the literature survey (Sect. 2), a series of machine learning approaches for the detection of cancer were analysed. Imaging datasets, biological and clinical data, and EHRs were primarily employed as the initial input source when developing cancer detection algorithms. This procedure involved a few preprocessing steps. First, the input source was typically preprocessed at the beginning stages of the experiment to extract regions or features of interest. Next, the retrieved set of features were then applied to downstream machine learning classifiers for cancer prediction. In this work, as opposed to using imaging datasets, clinical and biological data or, EHRs as the starting input source, this work proposes to use raw DNA sequences as the only input source. Moreover, contrary to using statistical methods or advanced CNNs for data extraction and representation, this work proposes to use state-of-the-art sentence transformers namely: SBERT and SimCSE. As far as we are aware, these two sentence transformer models have not been applied for learning representations in cancer research. The learned representations will then be fed as input to machine learning algorithms for cancer prediction.

## Methods

### Data description

In this study, 95 samples from colorectal cancer patients and matched-normal samples from previous work [104] were analysed. Exon sequences from two key genes: *APC* and *ATM* were used. The full details of the exons that were used in this study is shown Tables 5 and 6. Table 7 shows the data distribution among the normal/tumor DNA sequences. Ethics approval was granted by the University of Pretoria EBIT Research Ethics Committee (EBIT/139/2020).

**Table 5:** Exon sequences extracted from the *APC* gene

Chromosome	Start	End	Gene
chr5	112,043,201	112,043,579	APC
chr5	112,073,555	112,073,622	APC
chr5	112,074,049	112,074,157	APC
chr5	112,090,569	112,090,722	APC
chr5	112,102,022	112,102,107	APC
chr5	112,102,885	112,103,087	APC
chr5	112,111,325	112,111,434	APC
chr5	112,116,486	112,116,600	APC
chr5	112,128,142	112,128,226	APC
chr5	112,136,975	112,137,080	APC
chr5	112,151,191	112,151,290	APC
chr5	112,154,662	112,155,041	APC
chr5	112,157,592	112,157,688	APC
chr5	112,162,804	112,162,944	APC
chr5	112,163,625	112,163,703	APC
chr5	112,170,647	112,170,862	APC

**Table 6:** Exon sequences extracted from the *ATM*

Chromosome	Start	End	Gene
chr11	108,093,558	108,093,913	ATM
chr11	108,098,321	108,098,423	ATM
chr11	108,098,502	108,098,615	ATM
chr11	108,099,904	108,100,050	ATM
chr11	108,106,396	108,106,561	ATM
chr11	108,114,679	108,114,845	ATM
chr11	108,115,514	108,115,753	ATM
chr11	108,117,690	108,117,854	ATM
chr11	108,119,659	108,119,829	ATM
chr11	108,121,427	108,121,799	ATM
chr11	108,122,563	108,122,758	ATM
chr11	108,123,543	108,123,639	ATM
chr11	108,124,540	108,124,766	ATM
chr11	108,126,941	108,127,067	ATM
chr11	108,128,207	108,128,333	ATM
chr11	108,129,712	108,129,802	ATM
chr11	108,137,897	108,138,069	ATM
chr11	108,139,136	108,139,336	ATM
chr11	108,141,790	108,141,873	ATM
chr11	108,141,977	108,142,133	ATM
chr11	108,143,258	108,143,334	ATM
chr11	108,143,448	108,143,579	ATM
chr11	108,150,217	108,150,335	ATM
chr11	108,151,721	108,151,895	ATM
chr11	108,153,436	108,153,606	ATM

**Table 7:** Data distribution

Gene	Total number of normal sequences	Total number of tumor sequences	Total
*Before SMOTE: chosen sampling strategy* = “*not majority’*’			
APC	305214	553563	858777
ATM	545113	610309	1155422
*After SMOTE: chosen sampling strategy* = “*not majority*”			
APC	553563	553563	1107126
ATM	610309	610309	1220618

### Data encoding

To encode the DNA sequences, state-of-the-art sentence transformers: Sentence-BERT [105] and SimCSE [105] were used. These transformers are explained in the next subsection.

#### Sentence-BERT

**Fig. 5:**
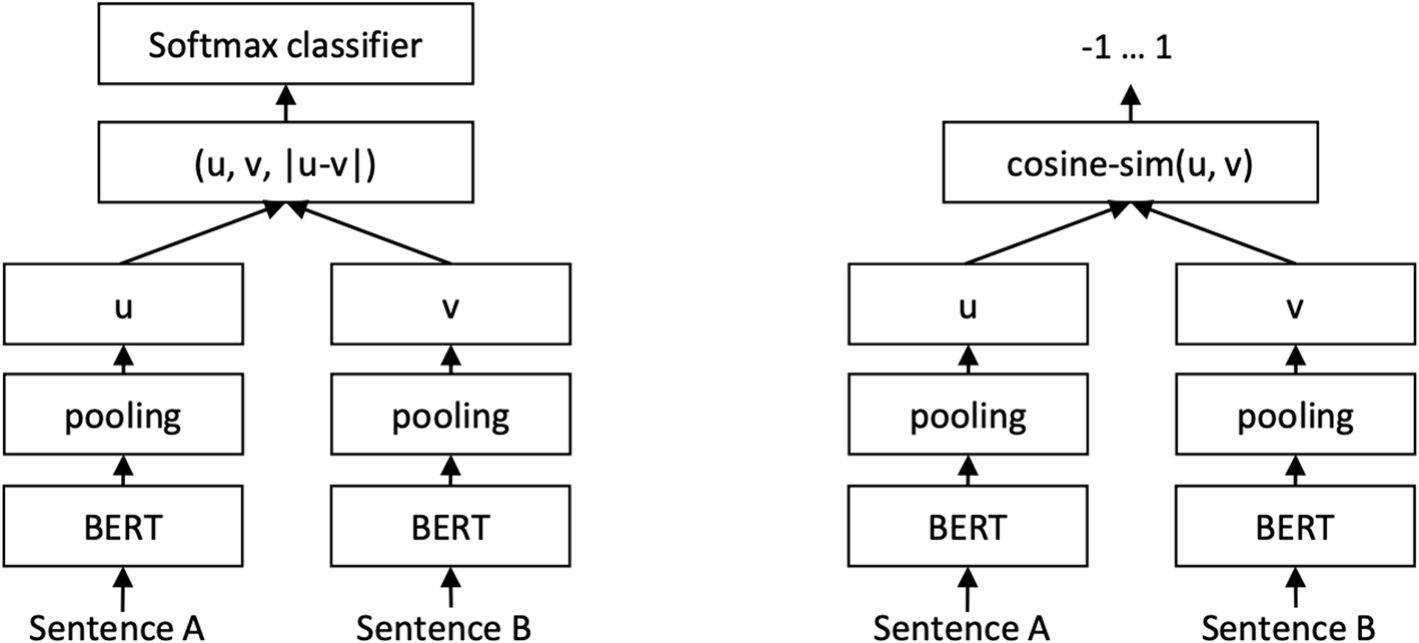
SBERT architecture with classification objective function (left) and the regression objective function (right) [105]

Sentence-BERT (SBERT) (Fig. 5) adapts the pretrained BERT [106] and RoBERTa [107] transformer network and modifies it to use a siamese and triplet network architectures to compute fixed-sized vectors for more than 100 languages. The sentence embeddings can then be contrasted using the cosine-similarity. SBERT was trained on the combination of SNLI data [108] and the Multi-Genre NLI dataset [109].

In its architecture, SBERT adds a default mean-pooling procedure on the output of the BERT or RoBERTa network to compute sentence embeddings. SBERT implements the following objective functions: classification objective function, regression objective function, and the triplet objective function. In the classification objective function, the sentence embeddings of two sentence pairs *u* and *v* are concatenated using the element-wise difference $\mid u-v \mid$ and multiplied with the trainable weight $W_{t} \epsilon {\mathbb {R}}^{3n *k}$:

1 $$\begin{aligned} o = softmax(W_{t}(u, v, \mid u-v \mid ) \end{aligned}$$

where *n* is the length or dimension of the sentence embeddings and *k* is the value of the target labels.

The regression objective function makes use of mean-squared-error loss as the objective function to compute the cosine-similarity between two sentence embeddings *u* and *v*.

The triplet objective function fine-tunes the network such that the distance between an anchor sentence *a* and a positive sentence *p* is smaller than the distance between sentence *a* and the negative sentence *n*.

Using the pretrained SBERT model: *all-MiniLM-L6-v2*, each DNA sequence was represented by a 384-dimensional vector.

#### SimCSE

**Fig. 6:**
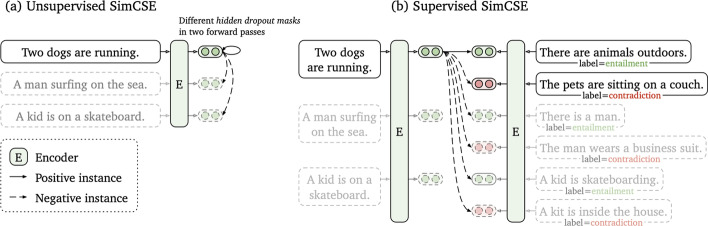
Unsupervised SimCSE (**a**) and supervised SimCSE (**b**) [110]

As with SBERT, Simple Contrastive Sentence Embedding (SimCSE) [110] (Fig. 6 is a transformer based model that modifies the BERT/RoberTa encoder to generate sentence embeddings. It uses a contrastive learning approach that aims to learn sentence representations by pulling close neighbours together and propelling non-neighbours. SimCSE comes in two learning forms: unsupervised and supervised SimCSE. In unsupervised SimCSE, the network is fine-tuned to predict the input sentence itself using dropout as noise then, the other sentences that are in the mini-batch are taken as negatives. In this case, dropout acts as a data augmentation method while previous [111, 112] methods have used word deletion, reordering, and substitution as a way of generating positive instances. In unsupervised SimCSE, an input sentence is fed twice to the encoder then, two embeddings with different dropout masks *z*, $z'$ are generated as output. The training objective for SimCSE is:

2 $$\begin{aligned} l_{i} = log \frac{e^{sim(h_{i}^{ z_{i}}, h_{i}^{z_{i}'})/\tau }}{\sum _{j=1}^{N} e^{sim(h_{i}^{z_{i}}, h_{j}^{z_{j}'})/\tau } } \end{aligned}$$

where *z* is the standard dropout mask that are found in Transformers and no additional dropout mask is added [110].

In supervised SimCSE, positive pairs are taken from the natural language inference (NLI) datasets and used to optimise the following equation:

3 $$\begin{aligned} l_{i} = -log \frac{e^{sim(h_{i}, h_{i}^{+})/\tau }}{\sum _{j=1}^{N} e^{sim(h_{i}, h_{j}^{+})/\tau } } \end{aligned}$$

where $\tau$ is a temperature hyperparamter and $sim(h_{1},h_{2})$ is the cosine similarity.

Using the unsupervised pretrained SimCSE model: *unsup-simcse-bert-base-uncased*, each DNA sequence was represented by a 768-dimensional vector.

### *K*-means clustering

The *k*-means clustering algorithm was used to visualize the sentence representations generated from SBERT and SimCSE in an unsupervised approach. The *k*-means algorithm divides the data points into *k* clusters where each data point is said to belong to the cluster centroid closest to it. Since the data consists of two types of documents (tumor vs. normal), the *k*-means algorithm was asked to find 2 clusters *n* and assign each DNA sequence to its closest centroid [113].

### Machine learning experiments

A total of three machine learning algorithms were used for classification: Light Gradient Boosting (LightGBM), eXtreme Gradient Boosting (XGBoost), and Random Forest (RF).

#### eXtreme gradient boosting (XGBoost)

eXtreme Gradient Boosting (XGBoost), is an efficient implementation of the gradient boosting algorithm. Gradient boosting belongs to a group of ensemble machine learning algorithms that be used to solve classification or regression problems. The ensembles are created from decision trees that are added one at a time to the ensemble, and fit to correct the classification error that were made by prior trees [114].

#### Light gradient boosting (LightGBM)

Light Gradient Boosting (LightGBM) machine is also a gradient boosting model that is used for ranking, classification, and regression. In contrast to XGBoost, LightGBM splits the tree vertically as opposed to horizontally. This method of growing the tree leaf vertically results in more loss reduction and provides higher accuracy while also being faster. LightGBM uses the Gradient-based One-Side Sampling (GOSS) method to filter out data instances for obtaining the best split value while XGBoost uses a pre-sorted and Histogram-based algorithm for calculating the best split value [115].

#### Random forest (RF)

Random forest (RF) is a supervised machine learning that is used in classification and regression tasks. It creates decision tress based on different samples and takes the majority vote for classification or average for regression. While XGBoost and LightGBM use a gradient boosting method, Random Forest uses a bagging method. The bagging method builds a different training subset from the training data with replacement. Each model is trained separately and the final result is based on a majority voting after consolidating the results of all the models [116].

#### Convolutional neural network (CNN)

Convolutional neural networks (CNNs) are a subset of neural networks that are frequently used to process speech, audio, and visual input signals. Convolutional, pooling, and fully connected (FC) layers are the three types of layers that are generally present in CNNs. The convolutional layer is the fundamental component of a CNN and is in charge of performing convolutional operations on the input before passing the outcome to the following layer. Then, the input is subjected to dimensionality reduction using pooling layers that reduces the number of parameters in the input. The FC layer uses a variety of activation functions, including the softmax activation function and the sigmoid activation function, to carry out the classification task using the features retrieved from the network’s prior layers [117, 118]. In this work, a three-layer CNN model with a sigmoid activation function will be supplied with the embedding features that were retrieved by SBERT and SimCSE sentence transformers. Due to computational limitations, the network will be trained over 10 epochs using the RMSprop optimizer and cross-validated over five folds.

### Performance evaluation metrics

To measure the performance of the machine learning models, the average performance of the models were reported using 5-fold cross validation and the following metrics were used: accuracy, precision, recall and F1 score. In Table 8, the definition of these metrics is provided.

**Table 8:** Performance evaluation metrics

Measure	Formula
Precision	tp/ (tp + fp)
Recall	tp/(tp+fn)
F1 score	2*(precision*recall)/(precision+recall)

*TP* True positives, *FP* False positives, *TN* True negatives, *FN* False negatives [119]

This section described the datasets used in the study as well as data representation methods and machine learning algorithms that were applied in this work. In the next section, the results of the applied methods are described.

## Results

### Visualizations

**Fig. 7:**
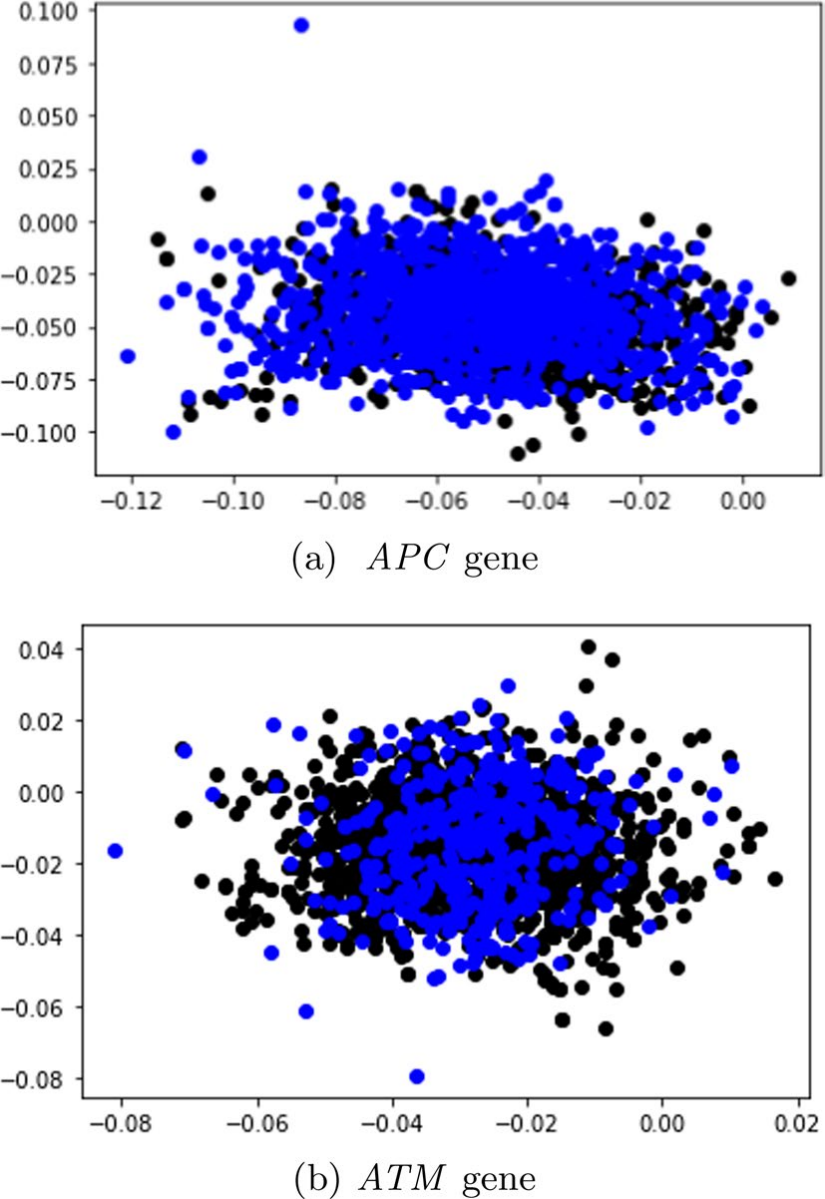
Visualisation of the SBERT documents with *k*-means clustering

**Fig. 8:**
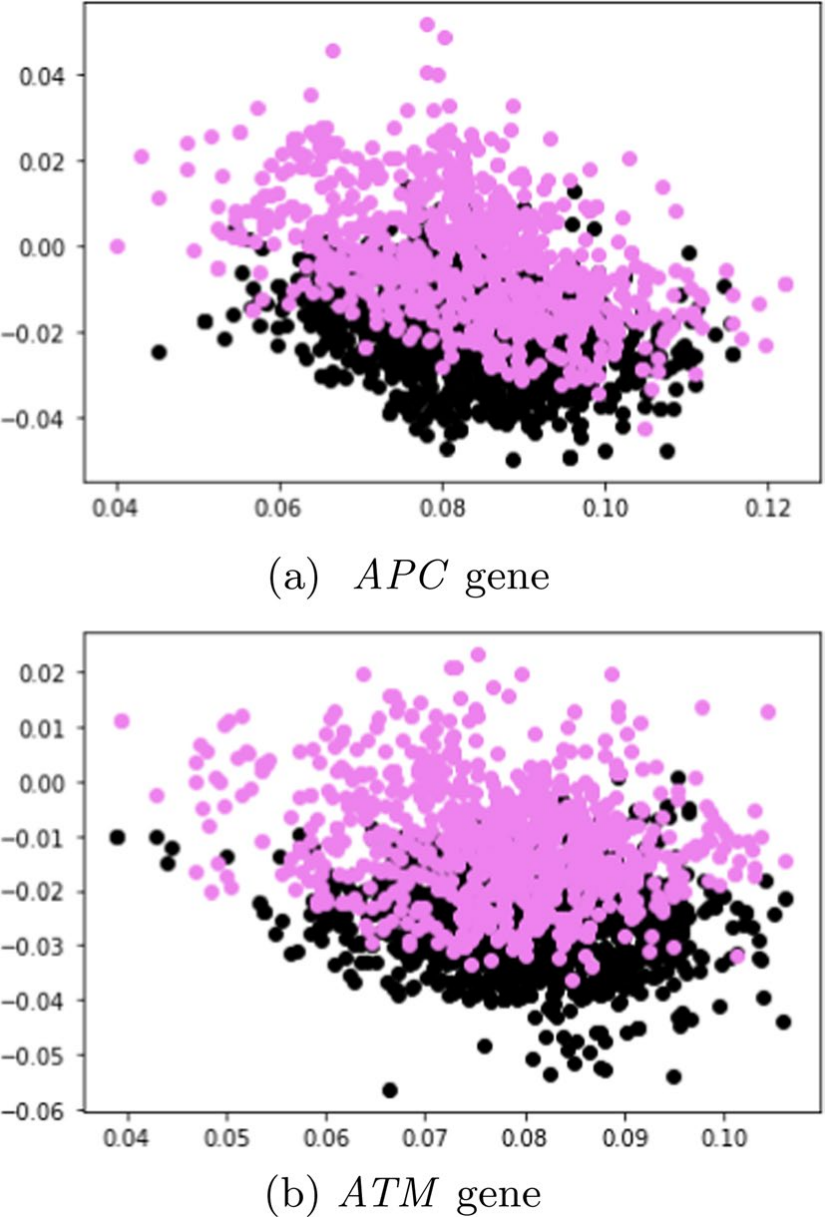
Visualisation of the SimCSE documents with *k*-means clustering

In this subsection, unlabeled data from SBERT and SimCSE representations were explored and visualized with the *k*-means clustering algorithm. The representations of the SBERT algorithm (Fig. 7) revealed more overlap between the data points in comparison to the representations of the SimCSE algorithm (Fig. 8). In the next subsection, machine learning models are evaluated to reveal if there is sufficient signal in the representations of the two sentence transformers that can discriminate between tumor and normal DNA sequences.

### Comparative performance of the machine learning results

#### SBERT before SMOTE

Table 9 presents the performance of the machine learning models on the *dev set* in terms of the average accuracy, averaged over the five folds using the SBERT representations. More performance metrics such as F1 score, recall, and precision are reported in the Additional file 1 (Appendix **A**).

**Table 9:** Development (dev) set accuracy (%) of the machine learning models

SBERT before SMOTE			SBERT after SMOTE	
	APC	ATM	APC	ATM
Random forest	65.9 ± 0.25	68.5 ± 0.68	51.4 ± 10.7	71.4 ± 1.16
XGBoost	62.5 ± 0.29	**73. ± 0.13**	62.5 ± 0.29	**73 ± 0.13**
LightGBM	64.9 ± 0.29	70.2 ± 0.64	**64.9± 0.29**	70.3 ± 0.64
CNN	**67.3 ± 0.04**	71.1 ± 2.84	47.0 ± 17.4	69.4 ± 5.2

SimCSE before SMOTE			SimCSE after SMOTE	
	APC	ATM	APC	ATM
Random forest	65.9 ± 0.15	73.2 ± 0.17	50.8 ± 10.9	**71.6 ± 1.47**
XGBoost	62.5 ± 0.65	73.7 ± 0.17	62.5 ± 0.65	68.8 ± 0.73
LightGBM	64.7 ± 0.29	**74. ± 0.18**	**64.7 ± 0.29**	70.7 ± 0.28
CNN	**67. ± 0.00**	73 ± 0.02	43. ± 0.17	71 ± 0.04

*APC*

Considering that the tumor DNA sequences belonging to the *APC* gene comprised of $\approx$ 64% of the data before SMOTE sampling, the machine learning models classified most sequences as positive (tumor); with the CNN achieving the best overall with the highest accuracy of 67.3 ± 0.04%.

*ATM*

In contrast to the data distribution of the *APC* gene before SMOTE sampling, the original data distribution of sequences from the *ATM* gene were relatively balanced as the tumor sequences comprised of 53% of the total data, and normal DNA sequences made up 47%. Moreover, as opposed to predicting nearly all sequences as positive, the machine learning models demonstrated an unbiased above-average performance as the highest performing model (XGBoost) achieved an accuracy of 73. ± 0.13 %.

#### SBERT after SMOTE

*APC*

**Fig. 9:**
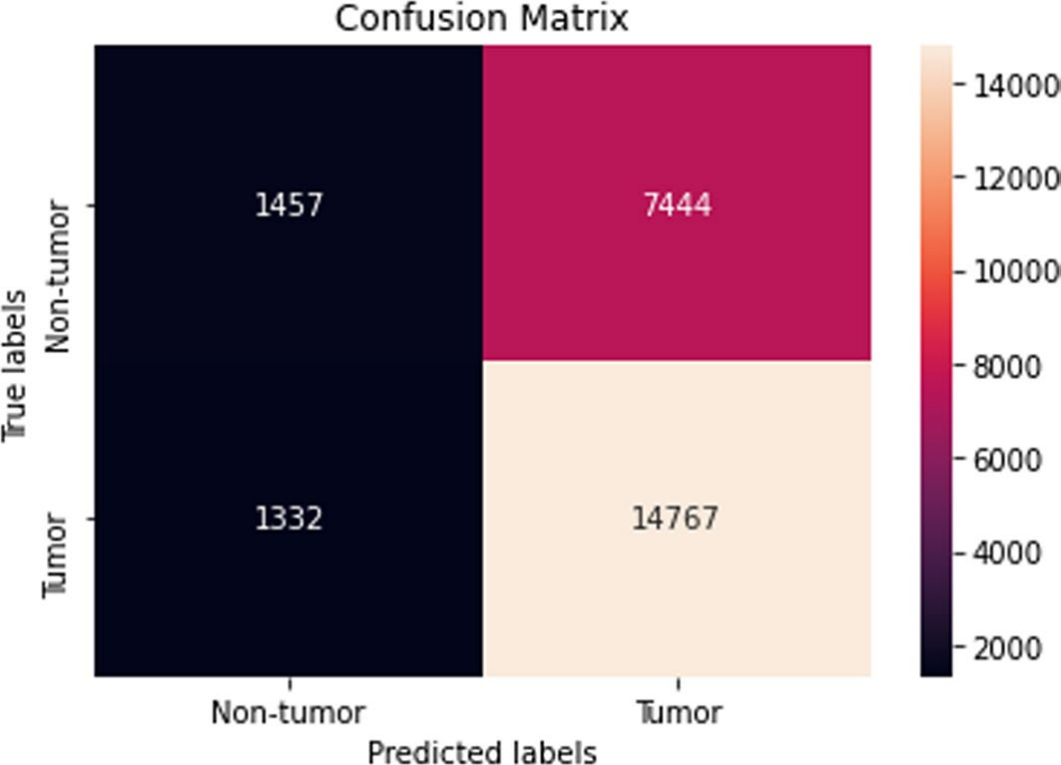
Confusion matrix of the LightGBM model using SBERT representations after SMOTE (dev set)

The performance of the majority of the machine learning classifiers after applying SMOTE remained consistent in that very little improvement or decline was observed. Moreover, while the CNN model previously obtained the highest overall accuracy before SMOTE oversampling, it performed the worst after applying SMOTE with a reported accuracy of 47. ± 17.4 %. Although biased, the LightGBM classifier reached the highest accuracy of 64.9 ± 0.29 %. Its confusion matrix is shown (Fig. 9).

*ATM*

**Fig. 10:**
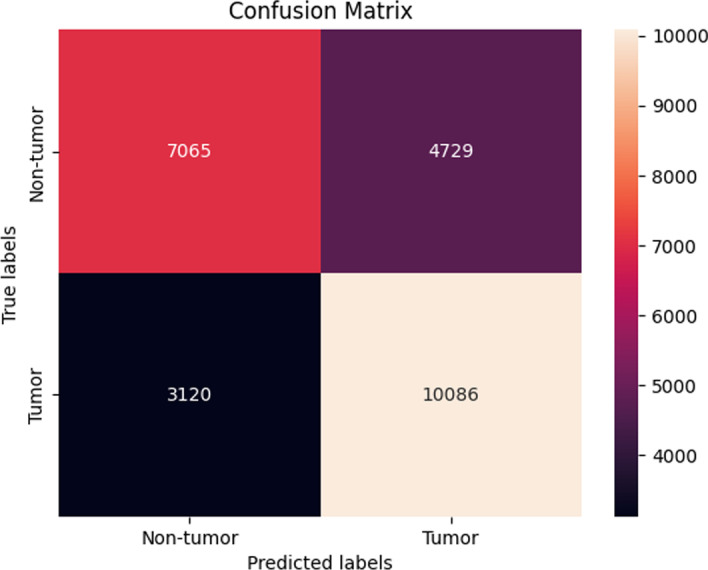
Confusion matrix of the XGBoost model using SBERT representations after SMOTE (dev set)

The same trend as seen in the previous Sect. 4.2.2 was also observed in this section with sequences from the *ATM* gene. Here, the performance of the machine learning models after SMOTE sampling was relatively similar to the performance of the machine learning models before SMOTE sampling as the XGBoost still maintained the best overall accuracy of 73. ± 0.13 % (Fig. 10).

#### SimCSE before SMOTE

Table 9 also presents the performance of the machine learning models in terms of the average accuracy, averaged over the five folds using the SimCSE representations. Supplementary performance metrics are reported (Additional file 1: Appendix A).

**Table 10:** Test set accuracy (%) of the machine learning models

SBERT before SMOTE			SBERT after SMOTE	
	APC	ATM	APC	ATM
Random forest	66.6 ± 0.36	73.3 ± 0.18	66.5 ± 0.33	**73.3 ± 0.16**
XGBoost	67.1 ± 0.40	73.2 ± 0.20	67.1 ± 0.40	**73.3 ± 0.20**
LightGBM	**67.4 ± 0.41**	73.3 ± 0.18	**67.4 ± 0.41**	**73.3 ± 0.18**
CNN	67.2 ± 0.42	**74. ± 0.12**	66.8 ± 0.42	70.71 ± 0.17

SimCSE before SMOTE			SimCSE after SMOTE	
	APC	ATM	APC	ATM
Random forest	66.5 ± 0.37	73.7 ± 0.12	66.6 ± 0.35	73.6 ± 0.14
XGBoost	67.1 ± 0.41	73.9 ± 0.12	67.1 ± 0.41	**75. ± 0.12**
LightGBM	**67.4 ± 0.41**	74.1 ± 0.20	**67.4 ± 0.41**	74.1 ± 0.20
CNN	67.4± 0.47	**75. ± 0.12**	67.3 ± 0.46	73.3 ± 0.14

*APC*

In this experimental setting, the performance of the machine learning models with SBERT representations before SMOTE sampling was similar to the performance of the models with SimCSE representations before SMOTE sampling. Here, the CNN achieved the best accuracy of 67. ± 0.0 %.

*ATM*

A similar pattern as in the previous Sect. (*APC*, SimCSE before SMOTE) was also detected in this setting when using sequences from the *ATM* gene in that the performance of the SimCSE models were almost similar to the performance of the SBERT models (before SMOTE) with slight improvement. The LightGBM model achieved the highest accuracy of 74. ± 0.18 % which was an improvement in accuracy of approximately 4 %.

#### SimCSE after SMOTE

*APC*

**Fig. 11:**
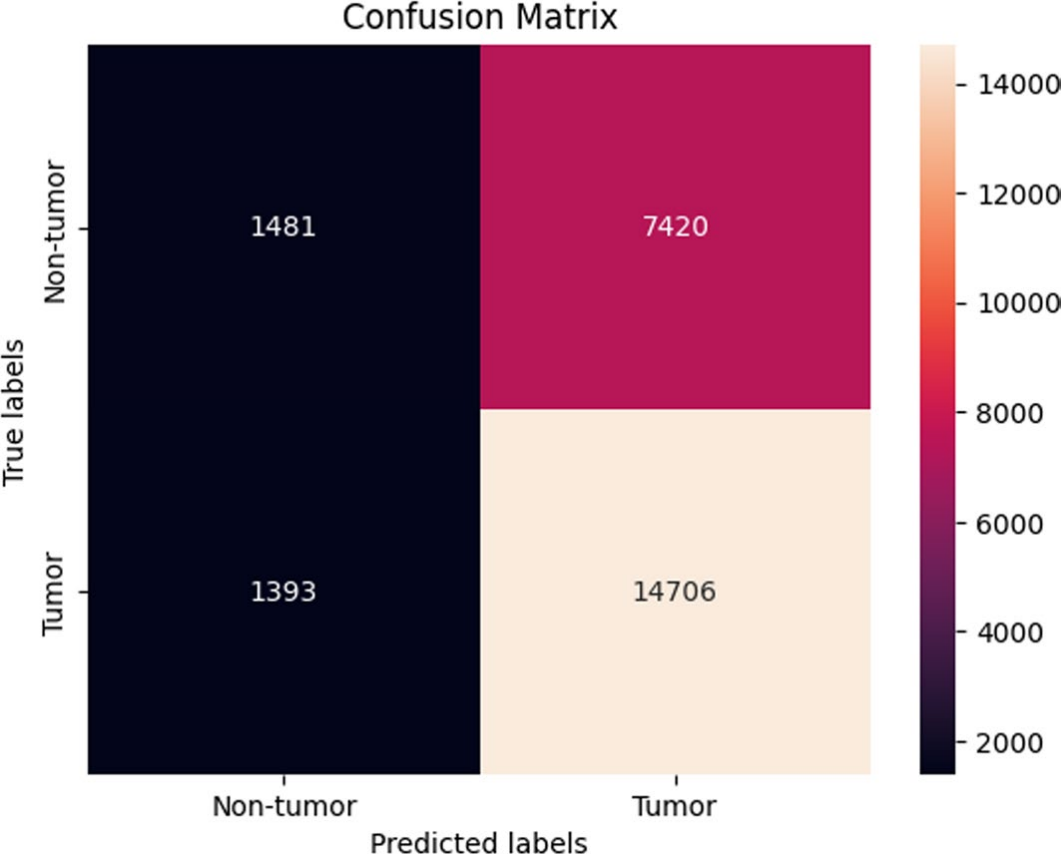
Confusion matrix of the LightGBM model using SimCSE representations after SMOTE (dev set)

The LightGBM model achieved the highest accuracy of 64.7 ± 0.29 (Fig. 11), which was indistinguishable to the performance reported before SMOTE oversampling.

**Fig. 12:**
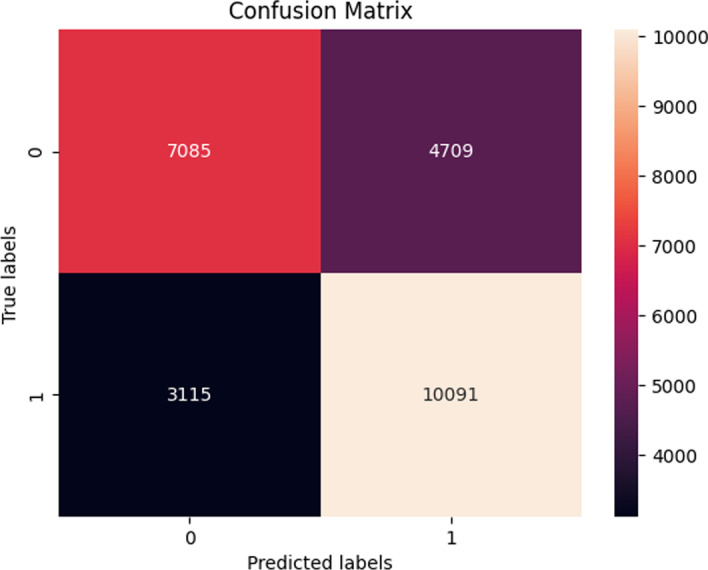
Confusion matrix of the Random forest model using SimCSE representations after SMOTE (dev set)

*ATM* In this final experimental setting, the results demonstrated a consistent performance before SMOTE sampling and after SMOTE sampling. The highest performing model was the Random forest model as it achieved an average accuracy of 71.6 ± 1.47 % (Fig. 12).

In Table 10, the experiments were repeated on an additional unseen test set. Overall, the machine learning models demonstrated a slight increase in the accuracy as the highest performing model, XGBoost, achieved an average accuracy of 75. ± 0.12 % using SimCSE representations from the *ATM* gene.

## Discussion

This paper provided a literature review of how cancer has been detected using various machine learning methods. Additionally, this work developed machine learning models that detect cancer using raw DNA sequences as the only input source. The DNA sequences were retrieved from matched tumor/normal pairs of colorectal cancer patients as described by previous work [104]. For data representation, two state-of-the-art sentence transformers were proposed: SBERT and SimCSE. To the best of our knowledge, these two methods have not been used to represent DNA sequences in cancer detection problems using machine learning. In summary of the results, we note that using SimCSE representations only marginally improved the performance of the machine learning models.

The ability to detect cancer by relying on human DNA as the only input source to a learning algorithm was one of the significant contributions of this work. We acknowledge that similar research investigating the role that the DNA plays in various cancer types has been conducted in the past. In contrary, the way the DNA was represented for the learning algorithms in our work is different from that in earlier research. An example would be work performed by [120] that used cell-free DNA (cfDNA) data from shallow whole-genome sequencing to uncover patterns associated with a number of different cancers including Hodgkin lymphoma, diffuse large B-cell lymphoma, and multiple myeloma. This study used PCA transformed genome-wide coverage features and applied them as input to a support vector algorithm to predict cancer status rather than employing sentence transforms for data representation as was done in our study. Another study [121] also used cfDNA sequences to predict cancer tissue sequences from healthy ones. In this work, reads from hepatocellular carcinoma (HCC) patients and healthy individuals were integrated with methylation information and then, a deep learning model was created to predict the reads that originated from a cancer tissue. The deep learning model consisted of a 1-d CNN followed by a maxpooling layer, a bi-directional LSTM, a 1-d CNN, and three dense layers. To represent the cfDNA sequences and methylation information, the variables were encoded into a one-hot encoded matrix that was then provided as input to the deep learning model for classification. Different from relying on raw DNA or cfDNA data to develop cancer detection frameworks, a study [122] consolidated methods from variant calling and machine learning to develop a model that detects cancers of unknown primary (CUP) origin which account for approximately 3% of all cancer diagnoses. This work employed whole-genome-sequencing-based mutation features derived from structural variants that were generated through variant calling and fed them as input to an ensemble of random forest binary classifiers for the detection of 35 different cancers.

## Limitations of the study

The machine learning experiments were only performed on two key genes: *APC* and *APC*, therefore it would have been interesting to see how the models generalize across various genes. The common disadvantage of conducting the experiments on multiple genes or whole genome sequencing data is that they require more computational resources which have a direct impact on cost. Another limitation of this work is that only two pretrained models were used for generating the sentence representations. Since there are several other pretrained models that are publicly available to choose from, some pretrained models were slower to execute than others hence a decision was made to focus on pretrained models that provided fast execution.

## Conclusion

This article reviewed the literature and demonstrated how various machine learning techniques have been used to identify cancer. Given that they are the most common malignancies worldwide, this work placed a special emphasis on four cancer types: lung, breast, prostate, and colorectal cancer. Then, a new method for the identification of colorectal cancer employing SBERT and SimCSE sentence representations was presented. Raw DNA sequences from matched tumor/normal pairs of colorectal cancer served as the sole input for this approach. The learned representations were then provided as input to machine learning classifiers for classification. In light of the performance of the machine learning classifiers, XGBoost was found to be the best performing classifier overall. Moreover, using SimCSE representations only marginally improved the classification performance of the machine learning models.

## Supplementary Information

**Additional file 1.** Appendix A.

## Data Availability

The data can be accessed at the host database (The European Genome-phenome Archive at the European Bioinformatics Institute, accession number: EGAD00001004582 Data access).
