# Supplementary material for: A review and comparative study of cancer detection using machine learning: SBERT and SimCSE application
Source: BMC Bioinformatics. 2023 Mar 23;24:112. doi: 10.1186/s12859-023-05235-x (PMC10037872; doi:10.1186/s12859-023-05235-x)
Supplement: Supplementary file 1 — Additional file 1. Appendix A. [file 12859_2023_5235_MOESM1_ESM.docx]

1 Appendix A

Table S1: Dev set F1 scores (%) of the machine learning models

| **SBERT before SMOTE** |  |  | **SBERT after SMOTE** |  |
| --- | --- | --- | --- | --- |
|  | **APC** | **ATM** | **APC** | **ATM** |
| **Random forest** | 77.7 *±* 0.11 | 71.5 *±* 0.72 | 48.8 *±* 24.4 | 74.5 *±* 2.65 |
| **XGBoost** | 74.2 *±* 0.29 | 78.2 *±* 0.09 | 74.2 *±* 0.27 | 78.2 *±* 0.09 |
| **LightGBM** | 77.1 *±* 0.20 | 74.8 *±* 0.64 | 77.1 *±* 0.21 | 74.8 *±* 0.64 |
| **CNN** | 79.2 *±* 0.07 | 74.8 *±* 4.47 | 47.7 *±* 25.1 | 69.1 *±* 11.15 |
| **SimCSE before SMOTE** |  |  | **SimCSE after SMOTE** |  |
|  | **APC** | **ATM** | **APC** | **ATM** |
| **Random forest** | 77.6 *±* 0.11 | 77.9 *±* 0.20 | 48.2 *±* 24.1 | 75.1 *±* 2.89 |
| **XGBoost** | 74.2 *±* 0.46 | 79.1 *±* 0.14 | 74.2 *±* 0.46 | 72.4 *±* 0.68 |
| **LightGBM** | 76.9 *±* 0.23 | 79.5 *±* 0.13 | 76.9 *±* 0.23 | 75.4 *±* 0.29 |
| **CNN** | 79. *±* 0.00 | 79 *±* 0.02 | 40. *±* 0.22 | 71. *±* 0.10 |

Table S2: Test set F1 scores (%) of the machine learning models

| **SBERT before SMOTE** |  |  | **SBERT after SMOTE** |  |
| --- | --- | --- | --- | --- |
|  | **APC** | **ATM** | **APC** | **ATM** |
| **Random forest** | 78.2 *±* 0.27 | 78.2 *±* 0.19 | 78.2 *±* 0.31 | 78.2 *±* 0.18 |
| **XGBoost** | 79.2 *±* 0.29 | 78.5 *±* 0.19 | 79.2 *±* 0.29 | 78.5 *±* 0.19 |
| **LightGBM** | 79.7 *±* 0.31 | 78.8 *±* 0.19 | 79.7 *±* 0.31 | 78.8 *±* 0.19 |
| **CNN** | 79.1 *±* 0.34 | 79.1 *±* 0.14 | 78.6 *±* 0.34 | 74.4 *±* 0.17 |
| **SimCSE before SMOTE** |  |  | **SimCSE after SMOTE** |  |
|  | **APC** | **ATM** | **APC** | **ATM** |
| **Random forest** | 78.2 *±* 0.30 | 78.6 *±* 0.17 | 78.2 *±* 0.31 | 78.6 *±* 0.15 |
| **XGBoost** | 79.3 *±* 0.29 | 79.4 *±* 0.11 | 79.3 *±* 0.29 | 79.4 *±* 0.11 |
| **LightGBM** | 79.7 *±* 0.31 | 79.7 *±* 0.22 | 79.7 *±* 0.31 | 79.7 *±* 0.22 |
| **CNN** | 79.4 *±* 0.36 | 80.2 *±* 0.15 | 76.7 *±* 5.19 | 78.1 *±* 0.20 |

Table S3: Dev set Recall scores (%) of the machine learning models

| **SBERT before SMOTE** |  |  | **SBERT after SMOTE** |  |
| --- | --- | --- | --- | --- |
| **Random forest** | **APC**  91.8 *±* 0.37 | **ATM**  76.1 *±* 1.30 | **APC**  43.4 *±* 21.8 | **ATM**  84.7 *±* 2.27 |
| **XGBoost** | 83.6 *±* 0.82 | 91.4 *±* 0.05 | 83.6 *±* 0.82 | 91.4 *±* 0.05 |
| **LightGBM** | 91.8 *±* 0.45 | 83.8 *±* 1.35 | 91.8 *±* 0.45 | 83.8 *±* 1.35 |
| **CNN** | 99.0 *±* 0.65 | 84.9 *±* 11.24 | 46.0 *±* 26.5 | 78.7 *±* 19.6 |
| **SimCSE before SMOTE** |  |  | **SimCSE after SMOTE** |  |
|  | **APC** | **ATM** | **APC** | **ATM** |
| **Random forest** | 91.8 *±* 0.35 | 89.8 *±* 0.39 | 42.7 *±* 21.4 | 86.1 *±* 2.18 |
| **XGBoost** | 83.6 *±* 0.94 | 93.8 *±* 0.35 | 83.6 *±* 0.94 | 77.4 *±* 1.02 |
| **LightGBM** | 91.2 *±* 0.59 | 95.6 *±* 0.14 | 91.2 *±* 0.59 | 85.2 *±* 0.70 |
| **CNN** | 99. *±* 0.00 | 95 *±* 0.07 | 34. *±* 0.21 | 82. *±* 0.17 |

Table S4: Test set Recall scores (%) of the machine learning models

| **SBERT before SMOTE** | **APC** | **ATM** | **SBERT after SMOTE APC** | **ATM** |
| --- | --- | --- | --- | --- |
| **Random forest** | 93. *±* 0.27 | 90.5 *±* 0.14 | 93. *±* 0.34 | 90.5 *±* 0.10 |
| **XGBoost** | 97.5 *±* 0.22 | 92.4 *±* 0.17 | 97.5 *±* 0.22 | 92.4 *±* 0.17 |
| **LightGBM** | 99.4 *±* 0.08 | 94. *±* 0.11 | 99.4 *±* 0.08 | 94. *±* 0.11 |
| **CNN** | 98.1 *±* 0.06 | 95.4 *±* 0.10 | 96.5 *±* 0.08 | 82.6 *±* 0.07 |
| **SimCSE before SMOTE** |  |  | **SimCSE after SMOTE** |  |
|  | **APC** | **ATM** | **APC** | **ATM** |
| **Random forest** | 93.1 *±* 0.14 | 91.6 *±* 0.16 | 93.1 *±* 0.25 | 91.5 *±* 0.22 |
| **XGBoost** | 97.6 *±* 0.17 | 94.8 *±* 0.20 | 97.6 *±* 0.17 | 94.8 *±* 0.21 |
| **LightGBM** | 99.3 *±* 0.08 | 96. *±* 0.21 | 99.3 *±* 0.08 | 96. *±* 0.21 |
| **CNN** | 99.2 *±* 0.06 | 98.7 *±* 0.06 | 98.6 *±* 0.03 | 92.2 *±* 0.22 |

Table S5: Dev set Precision scores (%) of the machine learning models

| **SBERT before SMOTE** | **APC** | **ATM** | **SBERT after SMOTE APC** | **ATM** |
| --- | --- | --- | --- | --- |
| **Random forest** | 67.3 *±* 0.08 | 68.2 *±* 0.50 | 56.1 *±* 28.1 | 66.9 *±* 5.24 |
| **XGBoost** | 66.6 *±* 0.27 | 68.3 *±* 0.15 | 66.6 *±* 0.27 | 68.3 *±* 0.15 |
| **LightGBM** | 66.5 *±* 0.20 | 67.6 *±* 0.52 | 66.5 *±* 0.20 | 67.6 *±* 0.52 |
| **CNN** | 66.5 *±* 0.20 | 68.2 *±* 0.48 | 53.3 *±* 26.9 | 64.6 *±* 7.3 |
| **SimCSE before SMOTE** |  |  | **SimCSE after SMOTE** |  |
|  | **APC** | **ATM** | **APC** | **ATM** |
| **Random forest** | 67.3 *±* 0.06 | 68.9 *±* 0.16 | 55.7 *±* 27.9 | 66.7 *±* 5.36 |
| **XGBoost** | 66.6 *±* 0.16 | 68.3 *±* 0.19 | 66.6 *±* 0.16 | 68. *±* 0.71 |
| **LightGBM** | 66.4 *±* 0.19 | 68. *±* 0.18 | 66.4 *±* 0.19 | 67.7 *±* 0.48 |
| **CNN** | 67. *±* 0.00 | 68. *±* 0.01 | 54. *±* 0.27 | 65. *±* 0.07 |

Table S6: Test set Precision scores (%) of the machine learning models

| **SBERT before SMOTE** | **APC** | **ATM** | **SBERT after SMOTE APC** | **ATM** |
| --- | --- | --- | --- | --- |
| **Random forest** | 67.4 *±* 2.14 | 68.8 *±* 0.25 | 67.5 *±* 0.44 | 68.8 *±* 0.21 |
| **XGBoost** | 66.7 *±* 0.47 | 68.3 *±* 0.25 | 66.7 *±* 0.47 | 68.3 *±* 0.25 |
| **LightGBM** | 66.5 *±* 0.45 | 67.9 *±* 0.24 | 66.5 *±* 0.45 | 67.9 *±* 0.24 |
| **CNN** | 66.1 *±* 0.45 | 68.2 *±* 0.24 | 66.8 *±* 0.45 | 68.5 *±* 0.29 |
| **SimCSE before SMOTE** |  |  | **SimCSE after SMOTE** |  |
|  | **APC** | **ATM** | **APC** | **ATM** |
| **Random forest** | 67.5 *±* 0.48 | 68.9 *±* 0.20 | 67.4 *±* 0.45 | 68.9 *±* 0.24 |
| **XGBoost** | 66.7 *±* 0.49 | 68.3 *±* 0.21 | 66.7 *±* 0.49 | 68.3 *±* 0.22 |
| **LightGBM** | 66.5 *±* 0.45 | 68.1 *±* 0.24 | 66.5 *±* 0.45 | 68.1 *±* 0.25 |
| **CNN** | 66.6 *±* 0.47 | 68.*±* 0.23 | 66.6 *±* 0.47 | 68.4 *±* 0.24 |
